# Correlation between controlled attenuation parameter values with SYNTAX score in patients with significant coronary artery disease

**DOI:** 10.1038/s41598-024-63792-4

**Published:** 2024-07-04

**Authors:** Jordan Sardjan, Cosmas Rinaldi Adithya Lesmana, Lusiani Rusdi, Juferdy Kurniawan, Evy Yunihastuti, Adityo Susilo, Rino Alvani Gani

**Affiliations:** 1 Department of Internal Medicine, Dr. Cipto Mangunkusumo National General Hospital, Medical Faculty University of Indonesia, Jakarta, Indonesia; 2 Division of Hepatobiliary, Department of Internal Medicine, Dr. Cipto Mangunkusumo National General Hospital, Medical Faculty University of Indonesia, Jakarta, Indonesia; 3 Division of Cardiology, Department of Internal Medicine, Dr. Cipto Mangunkusumo National General Hospital, Medical Faculty University of Indonesia, Jakarta, Indonesia; 4 Division of Allergy and Immunology, Department of Internal Medicine, Dr. Cipto Mangunkusumo National General, Hospital, Medical Faculty University of Indonesia, Jakarta, Indonesia; 5 Division of Tropical and Infectious Disease, Department of Internal Medicine, Dr. Cipto Mangunkusumo National General Hospital, Medical Faculty University of Indonesia, Jakarta, Indonesia

**Keywords:** Cardiology, Gastroenterology

## Abstract

Non-alcoholic fatty liver disease (NAFLD) is an emerging cause of chronic liver disease, with coronary artery disease (CAD) as the main cause of death in NAFLD patients. However, correlation between the severity of liver steatosis and coronary atherosclerosis is yet to be understood. Here we aim to explore the correlation between controlled attenuation parameter (CAP) values and SYNTAX (Synergy Between Percutaneous Coronary Intervention with Taxus and Cardiac Surgery) score in adult patients with significant CAD, defined as ≥ 50% stenosis of the left main coronary artery, or ≥ 70% stenosis of the other major coronary arteries. A cross-sectional study was conducted on 124 adult patients with significant CAD who underwent coronary angiography. Transient elastography with CAP was used to assess liver steatosis severity, resulting in a mean CAP value of 256.5 ± 47.3 dB/m, with 52.5% subjects had significant steatosis (CAP value of ≥ 248 dB/m). Median SYNTAX score was 22. A statistically significant correlation was observed between CAP value and SYNTAX score (r = 0.245, *p* < 0.0001). The correlation was more pronounced in patients with prior history of PCI (r = 0.389, *p* = 0.037). Patients with high-risk SYNTAX score (> 32) had the highest CAP value (285.4 ± 42.6 dB/m), and it was significantly higher than those with low-risk SYNTAX score (0–22), with a mean difference of 38.76 dB/m (*p* = 0.006). Patients with significant liver steatosis should undergo periodic CAD assessment and lifestyle modification, especially those with severe liver steatosis.

## Introduction

Non-alcoholic fatty liver disease (NAFLD), which is one of the aetiology of chronic liver disease (CLD), is characterized by the accumulation of ≥ 5% fat in the liver parenchymal tissue without a history of significant alcohol consumption^1^. NAFLD can progress to non-alcoholic steatohepatitis (NASH), liver cirrhosis (LC), and eventually lead to hepatocellular carcinoma (HCC) development^1^. The last few decades have witnessed a huge growth of NAFLD patients, including in Southeast Asia, especially Indonesia^2–5^. Coronary artery disease (CAD) is known as the main cause of death in NAFLD patients^6^. NAFLD is an independent risk factor for CAD, where NAFLD patients have a 1.68 folds higher risk of having CAD^7^. Transient elastography (TE) is one of the most validated non-invasive methods for hepatic fibrosis and steatosis staging. TE is rapid (less than 10 min), painless, has minimal complications, and can be performed at patients’ bedside^8^. Controlled attenuation parameter (CAP) is the tool available on the TE system (Fibroscan® by Echosens, Paris, France) for the quantification of liver steatosis as it assesses the ultrasound beam attenuation, which is directly related to liver fat content^9^. Increasing CAP value is associated with reduced risk for cardiovascular complications and mortality in individuals with type 2 diabetes mellitus (T2DM) and NAFLD^10^. Meanwhile in patients with significant CAD, the degree and score of the stenosis alone do not determine the choice of revascularization method as the extent of the lesion(s) and the suitability of the lesions for PCI or CABG also play a decisive role. Therefore, this study used SYNTAX (Synergy Between Percutaneous Coronary Intervention with Taxus and Cardiac Surgery) score to evaluate coronary artery complexity as it has been validated to assess the complexity of coronary atherosclerosis in patients with significant CAD^11^. However, to our knowledge, the correlation between CAP values and SYNTAX score in adult patients with significant CAD is yet to be known.

## Methods

### Study design and population

**Figure 1:**
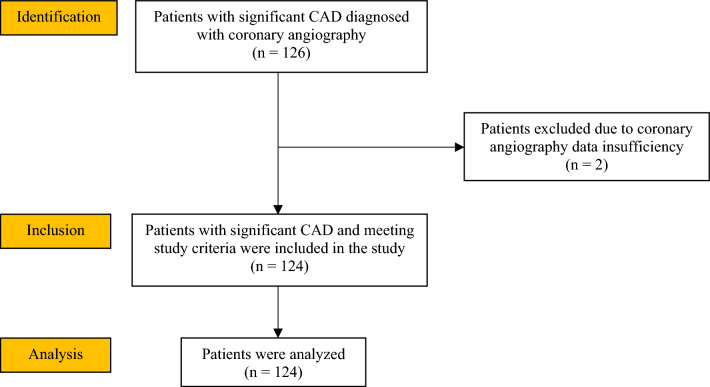
Study flow chart.

This was a cross-sectional study conducted at the Integrated Cardiac Care (PJT) Unit of Dr. Cipto Mangunkusumo National General Hospital (RSCM) from January to October 2023. The required sample size was calculated using a numerical correlation analysis formula. The alpha value used was five percent, with a beta of twenty percent, and the correlation estimate used was 0.25 based on prior study^12^. Therefore, we required a minimum sample size of 124 subjects. Two erroneous enrollments (patients initially reported as having significant CAD, but their subsequent coronary angiogram results were corrupted, hence their SYNTAX score could not be calculated) were excluded from the study (Fig. 1).

Inclusion criteria were adult patients who underwent coronary angiography at the catheterization laboratory of PJT RSCM, who were already proven to have significant CAD. We excluded patients with history of viral hepatitis, Human Immunodeficiency Virus (HIV), significant ascites, having a history of excessive alcohol consumption (> 21 drinks on average/week or > 30 g/day for men, and > 14 drinks on average/week or > 20 g/day for women), and those with a history of using drugs that can cause or increase steatosis (vitamin E 800 IU/day for 96 weeks, pioglitazone 30 mg/day for 96 weeks^13^, amiodarone cumulative dose of 290 ± 40.4 g in 37.6 months, tamoxifen 20 mg/day^14^, ursodeoxycholic acid 23–28 mg/kgBW/day for 18 months^15^).

### Clinical variables, measurements, and definitions

Medical interview, physical and laboratory examinations, and CAP measurement were conducted on selected patients who agreed to take part in the research. Smoking status were classified based on the definition by Centers for Disease Control and Prevention (CDC)^16^. Ex-smokers were defined as subjects who had smoked more than 100 cigarettes during their entire life and had not been smoking in the last 28 days. Patients who had smoked more than 100 cigarettes during their lifetime and whose last smoking activity were done in the last 28 days were classified as active smokers. Non-smokers were patients who had never smoked more than 100 cigarettes in their lifetime. Brinkman Index were calculated, by multiplying the number of cigarettes smoked per day with smoking duration in years. Patients with Brinkman Index of 0–199, 200–599, and ≥ 600 were classified as mild, intermediate, and heavy smoker respectively^17^. The diagnosis of T2DM were based on positive finding in any of these features (ADA 2023): (i) fasting plasma glucose (FPG) ≥ 126 mg/dL (fasting was defined as having zero calorie intake for at least 8 h), or (ii) random plasma glucose ≥ 200 mg/dL, or those with classical symptoms of diabetes or hyperglycaemic crisis, or (iii) 2 h plasma glucose ≥ 200 mg/dl during an oral glucose tolerance test (OGTT) with a 75-g glucose load, or (iv) A1C ≥ 6.5%^18^. Patients with history of T2DM or those on the needs of antidiabetic agents were also classified as diabetics. Hypertension were diagnosed when patients had history of hypertension, having the needs of antihypertensive agents, or those with resting blood pressure of ≥ 140/90 mmHg measured twice in two different arms^19^. Features of the metabolic syndrome according to the Adult Treatment Panel (ATP) III were recorded, including: (i) increased waist circumference (≥ 90 cm for men, ≥ 80 cm for women), (ii) increased blood pressure (≥ 130/85 mmHg), (iii) increased fasting blood glucose (≥ 100 mg/dL), (iv) low HDL cholesterol (< 40 mg/dL for men, < 50 mg/dL for women), and (v) high triglycerides (≥ 150 mg/dL)^20^. The metabolic syndrome was considered present when three of the five traits are found in the subject.

### Assessment of CAP value and SYNTAX score

Liver steatosis examination was carried out by using TE. TE is a non-invasive ultrasound-based examination that uses shear wave velocity to assess liver parenchyma. CAP, implemented in the FibroScan® device, measures the attenuation of the ultrasound beam as it passes through the liver to obtain steatosis quantification^9^. The technology is based on the evaluation of ultrasound signals acquired using a TE probe which is put on the skin surface overlying the liver (typically the 9th–11th intercostal space, on the midaxillary line). Upon pressing the button on the probe, a pulse wave is transmitted across the liver parenchyma. Shortly after the first wave, a second ultrasound wave is transmitted and the difference between the velocity of the two waves in the liver parenchyma is calculated. CAP is measured with a probe sized M (for general adult population) or XL (for overweight people) at 3.5 MHz frequency. CAP is calculated simultaneously with liver stiffness only if the measurements are valid. Measurement confidence is defined as: (1) obtaining 10 valid measurements or more, (2) interquartile range/median (IQR/med) less than 30% in elastography, and (3) success rate greater than 60%^21^. TE with CAP allows a rapid, reliable, reproducible, and non-invasive quantification of liver steatosis, with good intra- and interobserver levels of agreement. All patients had to fast for at least 3 h before the exam was conducted. TE was performed by two independent operators who were experienced (each operator had performed more than 100 examinations) in operating TE and interpreting its result. Both operators were blinded from patients’ metabolic status and coronary angiography results to keep the TE examination objective and unbiased. The average CAP values obtained from both operators were calculated and then analysed. Based on previous studies, we used a CAP cut-off value of 248 dB/m or higher to define subject with significant steatosis^22–25^.

Coronary angiography results were visualized by using Quantitative Coronary Angiography (QCA) software to provide better detail of each coronary artery lesions. Significant CAD was defined as ≥ 50% stenosis of the left main coronary artery, or ≥ 70% stenosis of the other major coronary arteries^26^. Patients’ SYNTAX scores were then calculated by senior interventional cardiologist who was blinded from patients’ metabolic status and CAP values.

### Statistical analysis

All statistical analyses were performed using the SPSS Statistics for Windows, version 27.0 (IBM, New York, USA). The data obtained were statistically analysed by two researchers. All measured variables and data with normal distribution were described as mean ± standard deviation (SD), while data with skewed distribution were presented with median and interquartile ranges. Difference between dichotomous variables were analysed by chi-square test. Mann–Whitney U test was used to analyse the differences between the two groups of continuous variables, and Levene's test was used to examine the homogeneity of the data. The Kruskal–Wallis and ANOVA tests were used to analyse the difference between more than two groups of independent variables. The Spearman correlation test was performed to evaluate the relationship between CAP value and SYNTAX score. A *p* value of < 0.05 defined statistical significance.

### Ethical approval and consent statement

This study obtained approval from The Ethics Committee of the Faculty of Medicine, Universitas Indonesia—RSCM (Approval number: 23-05-0586). Study was performed in accordance with the International Council for Harmonisation of Technical Requirements for Pharmaceuticals for Human Use Guideline for Good Clinical Practice (ICH-GCP). All enrolled patients had provided informed consent prior to their participation in this study.

## Results

### Basic characteristics of subjects

A total of 124 subjects were included in the final analysis. Two subjects were excluded from the study as their angiography results were inaccessible due to data errors. The baseline characteristics of the subjects can be seen in the Table 1 below.

**Table 1:** Characteristics of study participants.

Variables	Value (n = 124)
Gender, n (%)	
Male	104 (83.9)
Female	20 (16.1)
Age (years), mean (SD)	59.8 (11.11)
Smoking status, n (%)	
Non-smoker	55 (44.4)
Ex-smoker	48 (38.7)
Active smoker	21 (16.9)
Brinkman index, median (IQR)	120 (0–420)
Smoking intensity, (n = 69), n (%)	
Mild smoker (Brinkman index 0–199)	18 (26.1)
Moderate smoker (Brinkman index 200–599)	34 (49.3)
Heavy smoker (Brinkman index > 599)	17 (24.6)
BMI, mean (SD)	25.6 (3.53)
BMI category, n (%)	
Underweight (< 18.5 kg/m^2^)	34 (27.4)
Normal (18.5—< 23 kg/m^2^)	1 (0.8)
Overweight (23 – < 25 kg/m^2^)	21 (16.9)
Obesity (≥ 25 kg/m^2^)	68 (54.8)
Waist circumference, median (IQR)	92 (87–98)
Male, median (IQR)	92 (88–98)
Female, median (IQR)	90.5 (84–97)
Hypertension, n (%)	
Yes	117 (94.4)
No	7 (5.6)
Systolic blood pressure (mmHg), mean (SD)	126.98 (20.37)
Diastolic blood pressure (mmHg), mean (SD)	75.27 (10.92)
T2DM, n (%)	
Yes	69 (55.6)
No	55 (44.4)
Fasting blood glucose (mg/dL), median (IQR)	96.50 (86.25–108)
Random blood glucose (mg/dL), median (IQR)	125.5 (106.25–159.75)
A1C (%), (n = 84), median (IQR)	5.9 (5.42–7.3)
HDL cholesterol (mg/dL), mean (SD)	38.78 (10.83)
HDL cholesterol ≥ 40 mg/dL, n (%)	
Yes	55 (44.4)
No	69 (55.6)
LDL cholesterol (mg/dL), median (IQR)	109.5 (83–141.75)
LDL cholesterol < 70 mg/dL, n (%)	
Yes	13 (10.48)
No	111 (89.52)
Total cholesterol (mg/dL), median (IQR)	170 (142–209.25)
Triglyceride (mg/dL), median (IQR)	118.5 (82–161)
Metabolic syndrome, n (%)	
Yes	88 (70.97)
No	36 (29.03)
Creatinine (mg/dL), median (IQR)	1.2 (1–1.6)
eGFR (ml/minute/1.73 m^2^), mean (SD)	61.33 (28.01)
AST (U/L), median (IQR)	23 (17–35)
ALT (U/L), median (IQR)	20 (15–33)
History of percutaneous coronary intervention (PCI), n (%)	
Yes	29 (23.38)
No	95 (76.61)
Type of CAD, n (%)	
Acute coronary syndrome (ACS)	46 (37.1)
Chronic coronary syndrome (CCS)	78 (62.9)
Type of ACS, (n = 46), n (%)	
STEMI	27 (58.7)
NSTEMI	11 (23.9)
UAP	8 (17.4)
Number of coronary artery affected, n (%)	
Single-vessel	19 (15.3)
Multi-vessel	105 (84.7)

A 5:1 male to female ratio was obtained, with a mean age of 59.8 ± 11.1 years. Former and active smokers were found in 48 (38.7%) and 21 (16.9%) subjects, with a median Brinkman index of 120 (IQR 0-420). Subjects’ mean BMI was 25.6 ± 3.5 kg/m^2^, with 68 (54.8%) study participants were classified in the obesity group. The median waist circumference was 92 cm (IQR 88–98) for men and 90.5 cm (IQR 84–97) for women. This study found that 94.4% of the subjects were hypertensive with a relatively well-controlled blood pressure, and 55.6% were diabetics with a median A1C of 5.9% (IQR 5.4–7.3). A total of 69 (55.6%) subjects had low HDL cholesterol level, with a mean of 38.8 ± 10.8 mg/dL, and 111 (89.5%) subjects had yet to achieve LDL cholesterol target, with a median of 109.5 mg/dL (IQR 83–141.8). A relatively modest kidney function was observed, with a median creatinine level of 1.2 mg/dL (IQR 1–1.6), while median AST and ALT levels were relatively within the normal range. A total of 78 (62.9%) subjects presented with chronic coronary syndrome, with most patients (n = 105, 84.7%) were diagnosed with multivessel disease.

### CAP value, SYNTAX score, and their correlation

The main outcomes of this study were the CAP value, SYNTAX score, and the correlation between them (Table 2). We obtained mean CAP value 256.5 ± 47.3 dB/m, with 65 subjects (52.5%) had significant steatosis (CAP ≥ 248 dB/m). The median SYNTAX score was 22 (IQR 17–28).

**Table 2:** Main outcomes.

Variables	Value (n = 124)
CAP value (dB/m), mean (SD)	256.49 (47.28)
Significant steatosis, n (%)	
Yes	65 (52.5%)
No	59 (47.5%)
SYNTAX score, median (IQR)	22 (17–28)

Statistical analysis was performed by using Spearman correlation test, resulting in a significant positive linear correlation between the CAP value and the SYNTAX score (r = 0.245 and p < 0.0001) as presented in Table 3.

**Table 3:** Correlation between CAP value and SYNTAX score.

	SYNTAX score	
	Corellation coefficient (r)	*P* Value
CAP value	0.245	< 0.0001

### Secondary outcomes

We also analyzed subjects’ characteristics based on SYNTAX score stratification (Table 4). Patients were identified as low risk (0–22), moderate risk (> 22 to 32), and high risk based on their respective SYNTAX score^27^.

**Table 4:** Characteristics of subjects based on SYNTAX score stratification.

Variables	SYNTAX Score stratum			*P* Value
	0–22 (Low) (n = 63)	> 22 to 32 (Intermediate) (n = 43)	> 32 (High) (n = 18)	
Age (years), mean (SD)	58.13 (11.63)	62.37 (9.12)	59.50 (11.36)	0.154
Gender, n (%)				
Male	53 (84.1)	34 (79.1)	17 (94.4)	0.329
Female	10 (15.9)	9 (20.9)	1 (5.6)	
Brinkman index, median (IQR)	150 (0–420)	3 (0–360)	330 (0–480)	0.515*
T2DM, n (%)				
Yes	35 (55.6)	21 (48.8)	13 (72.2)	0.245
No	28 (44.4)	22 (51.2)	5 (27.8)	
Fasting blood glucose, median (IQR)	98 (89–109.5)	102 (84.5–113.7)	96 (86–190)	0.907*
Random blood glucose, median (IQR)	128 (109–164)	121 (107.3–157.8)	198 (102–248)	0.422*
A1C (%), (n = 84), median (IQR)	5.9 (5.3–7.4)	5.95 (5.4–7.1)	6.2 (5.8–8.3)	0.403*
Hypertension, n (%)				
Yes	59 (93.7)	41 (95.3)	17 (94.4)	0.933
No	4 (6.3)	2 (4.7)	1 (5.6)	
Systolic blood pressure, mean (SD)	126.7 (17.6)	130.5 (20.7)	119.6 (26.9)	0.158**
Diastolic blood pressure, mean (SD)	75.3 (9.9)	77 (11.7)	71 (11.9)	0.163**
Waist circumference (cm), median (IQR)	92 (87–96)	92 (88–98)	90.5 (88–95)	0.845*
BMI, mean (SD)	25.38 (3.58)	26.03 (3.42)	25.33 (3.69)	0.614
HDL cholesterol (mg/dL), mean (SD)	39.97 (11.72)	39.11 (9.65)	33.81 (9.29)	0.100
LDL cholesterol (mg/dL), median (IQR)	110 (81–155)	108 (85–137)	121.5 (84.5–140.25)	0.938*
Total cholesterol (mg/dL), median (IQR)	160 (135–231)	174 (148–200)	175.5 (124.5–196.75)	0.842*
Triglyceride (mg/dL), median (IQR)	118 (80–162)	128 (94–165)	116.5 (83–139.25)	0.591*
Metabolic syndrome, n (%)				
Yes	41 (65.08)	33 (76.74)	14 (77.78)	0.339
No	22 (34.92)	10 (23.26)	4 (22.22)	
History of PCI, n (%)				
Yes	20 (31.7)	7 (16.3)	2 (11.1)	0.075
No	43 (68.3)	36 (83.7)	16 (88.9)	
Type of CAD, n (%)				
ACS	26 (41.3)	14 (32.6)	6 (33.3)	0.619
CCS	37 (58.7)	29 (67.4)	12 (66.7)	
**CAP value, mean (SD)**	**246.67 (44.85)**	**258.77 (48.29)**	**285.43 (42.63)**	0.007**
Steatosis, n (%)				
Non-significant (CAP < 248 dB/m)	33 (52.4)	22 (51.2)	4 (22.2)	0.066
Significant (CAP ≥ 248 dB/m)	30 (47.6)	21 (48.8)	14 (77.8)	

*Kruskal–Wallis Test, ** One-way Anova Test.

Significant differences in CAP values between SYNTAX subgroups.

As shown in Table 4, CAP values increased in a stepwise manner as the SYNTAX score grading increased. There was a significant difference in CAP values between subjects with different grade of SYNTAX score (*p* = 0.007). A post-hoc analysis for mean CAP values difference between SYNTAX score subgroups was carried out as shown in Table 5 below. A significant difference of 38.76 dB/m in the mean CAP values between the low and high SYNTAX score group was obtained (*p* = 0.006). No significant differences were found in other characteristics.

**Table 5:** Post-hoc analysis of mean CAP value difference between SYNTAX score subgroups.

SS Category (A)	SS Category (B)	Mean CAP Value Difference	*P* Value
Low	Intermediate	− 12.10	0.552
	High	− 38.76	0.006
Intermediate	Low	12.10	0.552
	High	− 26.66	0.120
**High**	**Low**	**38.76**	**0.006**
	Intermediate	26.66	0.120

Significant differences in CAP values between SYNTAX subgroups.

### Correlation between CAP value and SYNTAX score based on history of prior PCI

As prior PCI could affect patients’ SYNTAX score, we also conducted further analysis to obtain the correlation between CAP value and SYNTAX score when subjects were classified based on the history of prior PCI as shown in Table 6. Compared to overall subjects, an increase in correlation between CAP values and SYNTAX score was found in the subgroup of subjects with a history of previous PCI (r = 0.389, *p* = 0.037). Meanwhile, in the subgroup who had never undergone PCI, the correlation between CAP values and SYNTAX score weakened and became insignificant (r = 0.194, *p* = 0.059).

**Table 6:** Correlation between CAP value and SYNTAX score based on history of prior PCI.

Parameter	SYNTAX Score			
	Subjects with history of PCI (n = 29)		Subjects without history of PCI (n = 95)	
	r	*P* value	r	*P* value
CAP Value	0.389	0.037	0.194	0.059

## Discussion

To our knowledge, this is the first study to investigate the correlation between CAP value and SYNTAX score in adult patients with significant CAD. In this cross-sectional study, patients were dominated by male with a ratio of 5:1 compared to female. The mean age of the subjects in our study was 59.8 ± 11.1 years. CAD prevalence increases at above 35 years of age in both male and female, with the lifetime risk of developing CAD for male and female at above 40 years of age is 49% and 32%, respectively^28^. Male gender and Southeast Asian ethnicity have also been established as non-modifiable risk factors for CAD^28^. Our study demonstrated that most subjects were in poor metabolic conditions, with 71% subjects had metabolic syndromes, 54.8% subjects were obese, 94.4% were hypertensive, 55.6% were diabetics, and 89.5% subjects had not achieved the desired LDL cholesterol target.

Liver steatosis and CAD have been found to be associated with various variables of metabolic syndrome, including insulin resistance, hypertension, dyslipidaemia, and abdominal obesity. Insulin resistance and dyslipidaemia, mainly increased LDL cholesterol and triglycerides, had been proven to cause vascular disruption and initiating the formation of atheroma plaque^29^. Meanwhile, the aforementioned metabolic abnormalities also contribute to increased accumulation of fat in the liver, resulting in liver steatosis^29,30^. With insulin resistance, the body is more prone to hyperlipidaemia, hyperinsulinemia, and hyperglycaemia, which progressively damage the vascular endothelium, leading to the release of reactive oxygen species (ROS) and a decrease in nitric oxide levels, thereby reducing coronary vasodilation capacity, decreasing blood vessel elasticity, promoting smooth muscle cell hyperplasia, macrophage apoptosis within atherosclerotic plaques, and LDL oxidation^31^. All of these pathological processes synergize in accelerating the process of atherosclerosis, including in the coronary arteries, which initiate the occurrence of CAD. It is evident that NAFLD shares a considerable common risk factors with CAD^32^. Therefore, the correlation between liver steatosis and CAD becomes of great significance, particularly in determining whether the severity of each entity is also correlated. Wong et al. (2011) found that fatty liver was independently associated with CAD (adjusted OR 2.31, 95% CI 1.46–3.64)^33^, and Mousa et al. (2022) revealed that NAFLD was significantly associated with CAD in a grade-dependent manner (*p* = 0.003)^12^. However, study by Vu et al. (2022) found that NAFLD was not strongly associated with coronary atherosclerosis^34^. The ultimate goal of our study was to broaden the current knowledge on the field of NAFLD and CAD, that the severity of liver steatosis (CAP value) and complexity of CAD (SYNTAX score) were positively correlated. We hope to raise more awareness, especially among patients with severe liver steatosis, to screen for CAD as early as possible, as they present with higher risk of developing more complex CAD.

In 2011, the CAP examination was introduced for the first time to evaluate and quantify the severity of liver steatosis, ten years after the initiation of transient elastography examination to assess hepatic fibrosis^35^. Based on previous studies, we used the optimal CAP cut-off value of 248 dB/m to define significant liver steatosis, and it was demonstrated that 52.5% of study subjects had significant steatosis^22–25^.

Based on our analysis, there was a positive correlation between CAP value and SYNTAX score (r = 0.245, *p* < 0.0001). Similar study was conducted by Mousa WA et al. in 2022, where they studied 125 patients suspected from having ischemic heart disease^12^. Angiography CT was used to confirm the diagnosis and degree of CAD, while abdominal CT was performed to confirm the presence of liver steatosis without assessing its degree. They obtained a similar result with our study, that liver steatosis was positively correlated with the degree of CAD (r = 0.250, *p* = 0.001)^12^.

Other variables that might affect the risk of CAD, such as age, gender, and various metabolic syndrome parameters, were also analysed based on subjects’ SYNTAX score (Table 4). Liver steatosis and CAD have been known to be associated with metabolic syndrome variables which often occur simultaneously, including insulin resistance, hypertension, dyslipidaemia, and abdominal obesity. Results in Table 4 showed only CAP score which had a statistically significant relationship with the degree of SYNTAX score (*p* = 0.007). Therefore, the existence of interactions or confounding from other variables can be excluded.

Additionally, a subgroup analysis was also performed based on patients’ prior history of PCI as a secondary outcome. A higher correlation coefficient was found in the subgroup who had undergone PCI compared to those who had not. This finding suggested that prior history of PCI modified the relationship between the CAP score and the SYNTAX score. A possible biological explanation is to look at various CAD risk factors which have been described in Table 4, but apparently there were no significant differences in other demographic and clinical variables between SYNTAX score subgroups. Thus, it was necessary to see whether there are other factors that have not been studied that influence the strength of their correlation, one of which was the duration of having liver steatosis and CAD^36,37^. Study by Arora et al. (2019) found that in 56 patients with type 2 diabetes and asymptomatic CAD, the severity of CAD was found to be increasing by > 40% in a period of seven years despite the fact that standard therapy or various efforts to reduce the risk of cardiovascular disease were given^36^. Meanwhile, a study by Wong et al. (2015) of 52 patients with NAFLD showed a significant increase in the degree of liver steatosis (grade 1 vs. grade 2, *p* = 0.046) over a period of 3 years^37^. From these studies, we could expect a time-dependent manner of progression in both liver steatosis and coronary atherosclerosis severity. To incorporate the duration of having both diseases as a factor that could affect the correlation between CAP value and SYNTAX score, we need to have a specific onset at which patients had the objective data to prove that was the very first time they were diagnosed with liver steatosis and CAD. Unfortunately, most Indonesian people have not been regularly doing medical check-ups, including our study subjects.

To our knowledge, this is the first study to examine the correlation between the severity of liver steatosis and coronary atherosclerosis complexity, both quantitatively. Hence, the parameters used (CAP and SYNTAX score) are objective, precise, and have been recommended by established guidelines (APASL, EASL, AHA, and ESC). This study used TE as one of the most validated non-invasive methods for liver steatosis diagnosis, and coronary angiography as the gold standard to diagnose CAD. Nevertheless, as this was a cross-sectional study, time was not accounted for in obtaining the correlation results. Therefore, a further study using cohort design is advocated in the future.

## Conclusion

There is a significant positive correlation between CAP value and SYNTAX score in adult patients with significant CAD.

### Supplementary Information

Supplementary Information.

## Data Availability

All data generated or analysed during this study are included in this published article and its Supplementary Information file.
